# Results from an online survey of adults with cystic fibrosis: Accessing and using life expectancy information

**DOI:** 10.1371/journal.pone.0213639

**Published:** 2019-04-12

**Authors:** Ruth H. Keogh, Diana Bilton, Rebecca Cosgriff, Dominic Kavanagh, Oliver Rayner, Philip M. Sedgwick

**Affiliations:** 1 Department of Medical Statistics, London School of Hygiene and Tropical Medicine, Keppel Street, London, United Kingdom; 2 Royal Brompton Hospital, Adult Cystic Fibrosis Centre, London, United Kingdom; 3 Cystic Fibrosis Trust, One Aldgate, Second floor, London, United Kingdom; 4 Institute for Medical and Biomedical Education, St George’s, University of London, Cranmer Terrace, London, United Kingdom; 5 South West London Elective Orthopaedic Centre, Epsom and St. Helier University NHS Hospitals, Epsom; Heidelberg University, GERMANY

## Abstract

Cystic fibrosis (CF) is the one of the most common inherited diseases. It affects around 10,000 people in the UK, and the median survival age is 47. Recent developments making use of longitudinal patient registry data are producing more detailed and relevant information about predicted life expectancy in CF based on current age and clinical measurements. The objective of this study was toconduct an online survey of adults with CF living in the UK using a web-based questionnaire to investigate: (i) if and how they access information on life expectancy; (ii) what they use it for; (iii) if they want more personalised information on life expectancy or the time until other milestones. The survey was advertised through the Cystic Fibrosis Trust using social media. There were 85 respondents, covering men (39%) and women (61%) aged 16–65. 75% had received information on life expectancy either from their CF care team (34%) or other sources (71%), the most common being the Cystic Fibrosis Trust website and research literature. Most people who received information found it to be beneficial and reported using it in a variety of ways, including to plan strategies for maintaining as best health as possible and to psychologically manage current health status. 82% of respondents were interested in more personalised information about their life expectancy, and participants also noted interest in other outcomes, including time to needing transplant or reaching a low level of lung function. Themes arising in text responses included the importance of good communication of information, the difficulty of relating general information to one’s own circumstances, and a desire for increased information on factors that impact on survival in CF. As an outcome from this work, research is underway to establish how information on life expectancy can be presented to people with CF in an accessible way.

## Introduction

Life expectancy for people with cystic fibrosis (CF) has increased considerably over recent decades due to improved treatments and care [1,2,3]. The estimated median survival age for babies born today with CF in the UK is 47 [4]. With increased life expectancy, it has become important to be able to predict prognosis. Numerous factors are associated with prognosis, including genotype, sex and clinical measurements such as pulmonary function. Several tools for prediction of survival in CF have been developed: for summaries see Buzetti et al [5], McCarthy et al [6], MacNeill [7]. Data from national patient registries provide the potential to develop prediction tools using longitudinal information from large samples with long-term follow-up and such tools have been developed using CF patient registry data in the US [8], Canada [9] and France [10]. Recent work using UK CF patient registry data has provided more detailed information on life expectancy for people with CF in the UK [11,12], including through a model providing ‘personalised’ predictions which take into account longitudinally collected clinical data available in the registry [13].

For survival prediction tools to be effective, it is necessary to ascertain if people with CF wish to have predicted life expectancy, and if so how it should be presented. However, there have been no prior studies to investigate this. To this end, we conducted an online survey targeted at people with CF aged 16 and over living in the UK to investigate: (i) if and how they access information on life expectancy; (ii) what they use it for; (iii) if they want more personalised information on life expectancy or the time until other milestones. This paper presents quantitative and qualitative results from the survey. The results are reported using the guidelines in the Checklist for Reporting Results of Internet E-Surveys (CHERRIES) [14].

## Materials and methods

### Design and development

The online survey consisted of a web-based questionnaire designed using Bristol Online Surveys (https://www.onlinesurveys.ac.uk) and accessed via a specific link. The target population was people with CF aged 16 and older living in the UK and the survey was available online for a two-week period (4–18 July 2016). This study was approved by the London School of Hygiene and Tropical Medicine (LSHTM) Research Ethics Committee (Reference 16138).

The questionnaire (S1 File; Table A in S2 File) was developed in close consultation with two patient advisors. It began with an explanation of who should complete it, how long it would take (around 10 minutes), where the results would be made available, and the anticipated value of the results for future research. Information about the researcher and a statement about funding were also given. The following filter statement was then used to clarify who should complete the questionnaire: *“The questionnaire is designed to be completed only by people with CF who are aged 16 or older*. *I kindly request that you do not complete this questionnaire if you are aged under 16 or do not have CF*.*”* Respondents were asked to confirm they were aged 16 or older using a tick box, and those reporting being under the age of 16 were directed to a message stating that the questionnaire was designed for people with CF aged 16 and older and requesting they do not continue. We followed guidelines for internet surveys, including institutional guidelines (LSHTM Standard Operating Procedure SOP-005-03, “Informed Consent for Research”), and used an implied informed consent model [14]. Following the above introductory information and age filter question, the following statement about ethics and consent was given: “*By completing this questionnaire you consent to your responses being used to produce a summary of the results*, *which will be published in a report*, *a summary information sheet*, *and articles in academic journals*. *The questionnaire is anonymous*. *Text responses will be summarised so that they do not enable individuals to be identified*. *No individual text responses will be reproduced directly in the results summary*. *This project has been approved by the London School of Hygiene and Tropical Medicine Research Ethics Committee*.” By confirmation of meeting the inclusion criteria, informed consent was presumed. Parental or guardian consent was not sought for minors who completed the survey (those aged 16 or 17) and this was approved by the London School of Hygiene & Tropical Medicine Research Ethics Committee. At the request of the Committee, we provided information on support available via CF Centres and the Cystic Fibrosis Trust Helpline at the start of the questionnaire, which was also repeated at the end of the questionnaire for those who completed it. Respondents could stop completing the questionnaire at any point and their results would not be saved. To help respondents to feel engaged with the research, they were asked at the end of the questionnaire to provide, if they wished, their email address, so that they could be sent information summarising the results of the survey (S3 File).

The questionnaire included 14 main questions and several sub-questions, with multiple choice or free text responses. The questions were under three subheadings: “About you”, “Whether and how you currently find information about life expectancy”, and “The potential for more personalised information on life expectancy”. Respondents were required to answer all questions, with the exception of some free text responses. Certain items were conditionally displayed based on responses to other items. Participants could not move on to the next page before all mandatory answers were completed. The majority of the multiple choice questions included an option of “Not sure”, “Prefer not to say”, or “Other”. Where “Other” was allowed we also enabled additional information to be added in a free text box. The questions were displayed over 3 pages. The introductory information, including filter statement, was displayed over 4 pages. Three further pages of information were presented at the end: these gave respondents the opportunity to provide their email address in order to receive a summary of the results, reminded them of support available, gave information about when and where the results would be made available, and thanked them for their participation. Participants could go back to review and change previous answers at any time before pressing “Finish” and there was an option on the last page to enable participants to download their responses.

All survey responses were collected by the Bristol Online Surveys system over encrypted SSL (Secure Sockets Layer) connections (https://www.onlinesurveys.ac.uk/help-support/bos-security/). No cookies were used. The response data were downloaded from the Bristol Online Surveys system by the main investigator (via a password), stored securely and are held in compliance with institutional requirements.

Pilot versions of the questionnaire were created and tested, including by patient representatives, to identify any issues arising with wording, question skips, typographical errors, and so on. We tested that the questionnaire worked on mobile phones and tablets as well as computers.

### Recruitment

We used an open survey that could be completed by anyone visiting the website, resulting in a convenience sample. The survey was open for two weeks (4^th^-18^th^ July 2016) and was advertised at the start of this period by the Cystic Fibrosis Trust using Facebook and Twitter via a link to an article about the investigator and research [15]. Reminders were placed after 1 week. The survey was also promoted by others on social media.

### Analysis

The response data were exported from the Bristol Online Surveys system as an excel spreadsheet. Data were subsequently manipulated and analysed using the R statistical software. Multiple choice responses were summarised by the numbers/percentages selecting each response. Free text responses were read and paraphrased so that no responses were directly reproduced, in accordance with information provided to respondents in the introduction to the questionnaire. Some very similar responses were combined, and the resulting combined responses are reported in their paraphrased form Free text responses were also summarised in themes, following guidance on thematic qualitative analysis [16]. The themes were data driven. The analysis included detailed familiarisation with the text responses, identification of features, and their consolidation into themes. This task was performed by RK, with input from all authors on the identification of key themes and their interpretation. It was hypothesized that responses may differ according to certain demographics. It was therefore investigated whether responses to the main questions (Questions 8, 9, 10, 12, 13, 14) differed by sex, age (under 30; 30+), and siblings status (no siblings with CF; siblings with CF). Tests of differences by demographic group were performed using Fisher’s exact test. Only completed questionnaires were analysed. The Bristol Online Surveys system did not record data from partially completed questionnaires.

### Participant feedback

The survey results were summarised in a pictorial information sheet, produced by a professional designer, and were publicised by the Cystic Fibrosis Trust through social media, in an online magazine article [17], and in a blog [18]. Respondents who provided an email address when they completed the questionnaire were emailed the information sheet directly.

## Results

### Response rates

The questionnaire was completed by 85 individuals. A total of 339 people accessed the first page of the survey: 216 stopped on page 1, which was the introduction to the questionnaire and research; a further 8 progressed through to the age filter question and to the ethics and consent statement (pages 2–3) but no further; 30 partially completed the questionnaire (page 5–9). Of those who viewed at least the first page, 34% (115/339) answered at least one question and 25% (85/339) completed the questionnaire. Of those who progressed beyond the introductory information and started the questionnaire, 74% (85/115) completed it and a further 10 answered all questions but did not submit their responses. The Bristol Online Survey system does not give the number of site visitors, but gave a response rate of 17%, indicating it was just over 500.

Most responses came on the first day it was released online and it appears that the use of reminder messages was successful in increasing the number of responses (Figure A in S2 File). Table B in S2 File gives information about exposure on social media. Most responses appear to have originated from the Facebook link. 56 of the 85 respondents (66%) expressed interest in seeing the results by providing an email address.

All subsequent analyses are based on the data from the 85 individuals who completed the questionnaire.

### Respondent characteristics

Respondent characteristics, based on data from the “About you” section of the questionnaire, are summarised in Table 1. 61% (n = 52) were female and the median age was 30 (range 16–65). Eight were not living in the UK, but all respondents were retained for subsequent analyses. The majority (69%, n = 59) were employed or studying. 52% (n = 44) were living with a partner, spouse or their children, and 34% (n = 29) were living with parents or other relatives. The majority of respondents had siblings (92%, n = 78), of whom 21% (n = 16) had one or more siblings with CF.

**Table 1:** Summary of demographic information from the questionnaire section “About you” (questions 2–7). Frequencies (N) and percentages (%) are presented except where specified (indicated by *).

Variable	N	%
**Sex**		
Male	33	38.8
Female	52	61.2
**Age**		
Range*	16–65	
Mean (SD)*	32.3 (10.1)	
Median (Interquartile range)*	30 (25–39)	
16–19	4	4.7
20–24	13	15.3
25–29	23	27.1
30–34	15	17.6
35–39	11	12.9
40–49	14	16.5
50+	5	5.9
**Employment status**		
Full-time employment	32	37.6
Part-time employment	11	12.9
Self-employed	9	10.6
Student	7	8.2
Homemaker	3	3.5
Disabled	12	14.1
Unemployed	9	10.6
Retired	2	2.4
**Living in the UK**		
Yes	77	90.1
No	8	9.4
**Living arrangements**		
Living at home with parents or other close family relatives or guardians	29	34.1
Living with a spouse or partner (including with children)	44	51.8
Living with friends or siblings	5	5.9
Living alone	7	8.2
**Has siblings**		
Yes	78	91.8
No	7	8.2
**Of those with siblings, description of siblings**		
Siblings without CF only	62	79.5
Siblings with CF only	11	14.1
Siblings both with CF and without CF	5	6.4

### Current access to and use of life expectancy information

Table 2 summarises responses to the questionnaire section on “Whether and how you currently find information about life expectancy”. A total of 64 respondents (75%) had obtained information on life expectancy, either from their CF care team (34%, n = 29) or other sources (71%, n = 60). The Cystic Fibrosis Trust website and research literature were the most the most commonly reported sources. Not everyone who sought information from their CF care team had received it.

**Table 2:** Summary of multiple choice questionnaire responses from the questionnaire section “Whether and how you currently find information about life expectancy” (Questions 8–10). Frequencies (N, out of 85 except where indicated) and percentages (%) are presented. The shaded areas indicate the sub-question was not applicable.

Question/Sub-question	Response	N	%	How beneficial did you find this information, in terms of whether you found the information interesting or useful to know? N (of the subtotal) (%)		
				Not at all beneficial	Somewhat beneficial	Very beneficial
8. Has your doctor/CF team ever provided you with information about your life expectancy as part of your routine care?^1^	Yes	21	24.7	4 (19.0)	11 (52.4)	6 (28.6)
	No	56	65.9			
	Not sure	8	9.4			
9. Have you ever actively sought information about your life expectancy from your doctor/CF team?^1^	Yes, and I received some information from them	17	20.0	2 (11.8)	10 (58.9)	5 (29.4)
	Yes, but I did not receive any information from them	6	7.1			
	No	60	70.6			
	Not sure	2	2.4			
9b. For those who answered "No"/”Not sure”: Do you think there will be a time when you will want more information about your life expectancy and, if so, for what purposes? [Ordered by percentage who selected each option] [n = 62]	Perhaps: in making other life plans	28	32.9			
	Perhaps: to help plan strategies for maintaining as best health as possible (e.g. your exercise programme, physical activity schedules)	22	25.9			
	Perhaps: to help manage mentally/psychologically your current health status	21	24.7			
	Perhaps: to help make decisions or have discussions jointly with your CF specialist team on future treatments	19	22.4			
	Perhaps: just for general information	14	16.5			
	Perhaps: in planning your family	13	15.3			
	Perhaps: in choosing how you spend your leisure time	12	14.1			
	No	11	12.9			
	Perhaps: in planning meeting a partner	6	7.1			
	Perhaps: in planning your career path	4	4.7			
	Perhaps: in planning your education	1	1.2			
10. Have you ever actively sought information about your life expectancy from any of the following other sources? ^2^	Reports from the Cystic Fibrosis Trust/the Cystic Fibrosis Trust website	35	41.2	5 (14.3)	23 (65.7)	7 (20.0)
	Research literature	33	38.8	1 (3.0)	25 (75.8)	7 (21.2)
	Patient websites/forums	25	29.4	3 (12.0)	18 (72.0)	4 (16.0)
	Other internet sites	22	25.9	8 (36.4)	10 (45.5)	4 (18.2)
	Other people	7	8.2	0 (0)	5 (71.4)	2 (28.6)
	Other sources	7	8.2	0 (0)	2 (28.6)	5 (71.4)
	None of these	25	29.4			
10h. For those who answered "None of these": Why have you not sought information about your life expectancy? ^3^ [n = 25]	Because you feel you have received most or all of the information you would like from your doctor/CF team	4	16.0			
	Because you don’t want to know	8	32.0			
	Because you feel the information available will not be relevant and/or useful to you	12	48.0			
	Other	7	28.0			

^**1**^ By combining the responses to questions 8, 9, and 10 we find that 75.3% (n = 64) had obtained information on life expectancy either from their CF care team (34.1%, n = 29) or other sources (70.6%, n = 60), and 25 individuals had never sought information on life expectancy from sources other than their CF care team.

^2^ The phrasing in the sub questions 10a-10f was “How beneficial did you find this information, in terms of whether you found the information interesting or useful to know?”.

^**3**^ Of the 25 individuals who responded to question 10h, 4 chose both “Because you don’t want to know” and “Because you feel the information available will not be relevant and/or useful to you”, 1 chose both “Because you feel the information available will not be relevant and/or useful to you” and “Other”, and 1 chose both “Because you feel you have received most or all of the information you would like from your doctor/CF team” and “Because you feel the information available will not be relevant and/or useful to you”.

Table F in S2 File shows results separately by sex, age and siblings status. Overall, 21 (25%) respondents received information on life expectancy as part of routine care, of whom 10 were women (19% of women) and 11 were men (33% of men) (p-value for a difference: 0.016). People aged under 30 were more likely to have been provided with information on life expectancy as part of their routine care, but less likely to save sought information from their CF care team or other sources: these differences were not statistically significant. A greater proportion of those without CF siblings compared to those with CF siblings had actively sought information on life expectancy from their CF care team (no siblings with CF: 30%, siblings with CF: 13%) and from other sources (no siblings with CF: 35%, siblings with CF: 6%), though only the latter was statistically significant (p-value = 0.031).

People reported using information on life expectancy in various ways (Table 3), the most common being to plan strategies for maintaining as best health as possible and to psychologically manage current health status. Of 25 participants who had not sought information from any source, 7 provided further text responses, which are summarised in Table C in S2 File. Key themes for these respondents were that they viewed such information as negative and preferred to focus positively on living life rather than death, considered it was not relevant to them, and assumed that their life expectancy depends on unknown future developments in treatment. Another respondent commented that life expectancy is difficult to talk about.

**Table 3:** Summary of responses to Question 11 (“How do you use, or how have you used in the past, any information which you have learned about your life expectancy, either from your doctor/CF care team or from other sources?”) in section “Whether and how you current find information about life expectancy”. Frequencies (N, out of 85) and percentages (%) are presented and the rows are ordered by the percentage who selected each option. Respondents could select more than one response.

Response	N	%
To help plan strategies for maintaining as best health as possible (e.g. your exercise programme, physical activity schedules)	28	32.9
To help manage mentally/psychologically your current health status	28	32.9
In making other life plans	25	29.4
Just for general information	24	28.2
In planning your family	20	23.5
I have never received any information about my life expectancy	20	23.5
In planning your career path	15	17.6
Not much	12	14.1
In choosing how you spend your leisure time	10	11.8
In planning your education	9	10.6
To help make decisions or have discussions jointly with your CF specialist team on future treatments	8	9.4
In planning meeting a partner	5	5.9

Additional text responses were given by 18 individuals (summarised in Table D in S2 File) about other sources of information they had used or what they had found beneficial about the information they had accessed. Other sources mentioned included Wikipedia, Google, presentations, and the general media.Topics covered in the (paraphrased) responses included: life expectancy is an emotional topic for discussion with the CF care team and it can be easier to investigate it by yourself, although information online is ‘generic’ while the care team knows you; there is a desire for honest and balanced information on life expectancy and comments that generic information doesn’t apply easily to individuals, especially after reaching the current estimated median age of survival; terminology around life expectancy can be confusing; it is important to balance scientific information with information on individual experiences; CF affects people differently and it can be difficult to relate the information available to your own condition. One respondent recalled having only discovered by chance at a young age that life expectancy was lower for people with CF.

### Interest in personalised information

Table 4 summarises responses to the questionnaire section on “The potential for more personalised information on life expectancy”. Nearly three quarters of respondents (73%, n = 62) indicated interest in personalised information about life expectancy, and 82% (n = 70) expressed interest in personalised information indicating how they are doing relative to other people of the same age, even if they are not specifically interested in life expectancy. The numbers who would prefer to receive such information by themselves or via their doctor were similar. Overall, 54% (n = 46) of respondents reported interest in personalised information on reaching certain milestones. The most frequently mentioned were transplant (63%, n = 29), reaching certain levels of lung function (52%, n = 24), and acquisition of infections (28%, n = 13). Other milestones related to ability to work, quality of life, living independently, CF-related disease and hospitalisation.

**Table 4:** Summary of responses from the questionnaire section “The potential for more personalised information on life expectancy” (Questions 12–14). Frequencies (N, out of 85 except where indicated) and percentages (%) are presented.

Question/Sub-question	Response	N	%
12. Would you like to be able to access more personalised information about your life expectancy? The personalised information on which this is based could include, for example, your FEV1% predicted and how this is changing as you get older, your weight, the treatments you are using, whether you have received an organ transplant, as well as more intrinsic features such as your gender and your genetics.	Yes	62	72.9
	No	11	12.9
	Not sure	12	14.1
12a. For those answering "Yes" to Question 12: How do you think you would prefer to receive this information?^3^[n = 62]	Doctor only	25	40.3
	Myself only	20	32.3
	Both	17	27.4
13. One of the aims of my research is to provide more personalised information on your life expectancy which can be updated as you get older to take into account up-to-date information about your health status. Would you find such information useful as an indicator of how you are doing, including how you are doing relative to other people the same age as you (even if you are not specifically interested in your life expectancy)?	Yes	70	82.4
	No	8	9.4
	Not sure	7	8.2
13a. For those answering "Yes" to Question 13: How do you think you would prefer to receive this information?^3^[n = 70]	Doctor only	27	38.6
	Myself only	29	41.4
	Both	14	20.0
14. Would you be interested in how long it might be until you reach other milestones, in addition to or instead of your overall life expectancy? For example reaching a level of FEV1% predicted, having a transplant, or acquiring chronic pseudomonas.	Yes	46	54.1
	No	20	23.5
	Not sure	19	22.4
Summary of other milestones that people mentioned in response to Question 14^1^^,^^2^: “Would you be interested in how long it might be until you reach other milestones, in addition to or instead of your overall life expectancy? For example reaching a level of FEV1% predicted, having a transplant, or acquiring chronic pseudomonas.”	Transplant	29	63.0
	Reaching a certain level of lung function (30% FEV1 mentioned by several)	24	52.2
	Acquiring infections	13	28.3
	Work related issues: Stopping, reducing or changing work	6	13.0
	Reduction in quality of life (ability to do physical activity, shortness of breath, sex, living independently)	5	10.9
	Having to take certain treatments (insertion of ports, needing oxygen)	4	8.7
	Other CF related disease (diabetes, liver damage)	4	8.7
	Weight loss	3	6.5
	Fertility issues (time to conceiving or becoming a parent)	3	6.5
	Increased hospital admissions/pulmonary exacerbations	3	6.5

^1^ The responses were in text form. The categories shown in the table were derived based on reading of the responses. Some individuals mentioned more than one milestone.

^2^ Additional milestones mentioned by single individuals were: having surgeries, becoming resistant to some drugs, post-transplant survival, end-stage CF, and whether they would die before their parents.

^3^ The full responses given were: “At the clinic from my doctor/CF care team”, “By myself, for example via an online tool” and people could choose one or both of these. There was also an “Other” category but no one chose that.

A higher proportion of women than men were interested in more personalised information about their life expectancy (women: 75%, men: 70%) (Table F in S2 File), but were also more likely to say they were not sure (women: 19%, men: 6%), while men were more likely to say they did not want such information (women: 6%, men: 24%) (overall p-value = 0.019). A greater proportion of women reported an interest in more personalised information about life expectancy as an indicator of how they are doing (women: 90%, men: 70%) (p-value = 0.020). Younger people were more likely to be interested in personalised information on life expectancy (under 30: 78%, 30+: 69%) (p-value = 0.687) and in such information as an indicator of how they are doing (under 30: 93%, 30+: 73%) (p-value = 0.024). There was little difference between the siblings groups in terms of interest in personalised information.

### Text response themes

There were several places in the questionnaire where text responses could be given. Some of these were summarised above and in more detail in Tables C and D in S2 File. At the end of the questionnaire respondents were also asked: “Is there any information you would like to access about your life expectancy or about reaching other milestones which has not been covered here, and if so what?”. The responses are given in Table E in S2 File. These included comments about how clinical factors and use of medications relate to life expectancy. Another theme was interest in more information about disease progression, including CF-related diabetes related complications, post-transplant survival, and patterns of decline. Other respondents noted the difficulty of making life decisions and said that more information on outcomes could help with decision making. People also indicated an interest in comparing themselves with others with CF, in terms of their health status and how they go about their care. The importance of good communication of information on life expectancy was mentioned, including that it could be used positively as a motivator.

Across the three main questions where text responses were obtained (10g, 10h, 15) the following three main themes were identified:

*Communication of information*: There is a need for information on life expectancy to be communicated effectively and honestly, with a balance between scientific results and individual experiences. This is an emotional topic and it can be easier to investigate alone, although discussion with the CF care team can provide more personally relevant information.

*Personally relevant information*: It is difficult to relate generic information to oneself. More information on outcomes could help individuals face decisions relating to family, work, and finances. For some, it would be useful to compare themselves to others with CF in terms of health status and care routines.

*Improving understanding of CF*: Respondents were interested in more information about how life expectancy was affected by clinical factors, and how it might be improved by modifiable factors; the effects of current medications and prospects for future medications; and other outcomes including post-transplant survival and patterns of decline.

## Discussion

The aim of this survey was to investigate if and how adults with CF access information about life expectancy, how they use it, and whether they are interested in more personalised information. The following messages emerged:

1. Respondents typically received some information on life expectancy, with sources including their CF care team, the Cystic Fibrosis Trust and research literature; usually they found the information to be beneficial.
2. Respondents used information on life expectancy in various ways, including helping them manage their health status, plan health strategies and making life plans.
3. Information on life expectancy was viewed as negative by some respondents, suggesting scope for emphasising the positive aspects of such information.
4. Respondents were interested in personally tailored information on life expectancy, and about how their circumstances relate to others with CF. Respondents expressed interest in receiving information via both care teams and online sources.
5. Respondents were interested in other milestones, including quality-of-life, suggesting scope for the involvement of people with CF in informing research questions.
6. People with CF face difficult challenges in making plans. More information on outcomes and progression could be helpful.

This study was the first to investigate access to life expectancy information in people with CF. We have shown that an online approach is feasible for investigating this sensitive topic. The number of responses was much greater than would have been possible using face-to-face interviews. The response quality was high, with respondents giving detailed text responses. The qualitative summary of text responses was data-driven rather than being defined using pre-determined themes and responses were reviewed by all authors to avoid specific researcher bias. An online questionnaire was chosen over face-to-face interviews for several reasons. Previous research in the UK CF Registry Survey 2016 [19] suggested that online surveys were favourable in this population and would elicit a greater number of respondents; it avoided cross-infection, was more convenient for participants, and allowed participants to consider their answers without pressure. A limitation of this study is that respondents were self-selected and therefore may not be representative of the population of adults with CF. However, it is reassuring that a sizeable minority reported little interest in information on their life expectancy, suggesting that the questionnaire did not just attract people with particular interest in gaining more information on life expectancy. The respondents included both men and women across a wide age range. We compared the sex and age characteristics of the study population with that of the UK CF population aged 16 and over using data available in the 2016 UK Cystic Fibrosis Registry report [4]. The study population had a higher percentage of females (61% compared to 46%). They also tended to be slightly older, with a median age of 30, compared to 50% of those in the registry (aged 16 and over) being in age groups 16–19, 20–23 and 24–27. The number of respondents to the survey (85) was small relative to the underlying UK CF population, with the UK CF Registry recording data on 5851 individuals aged 16 and over in the year the survey was conducted (2016). However, recent online surveys conducted in the CF community would suggest it is difficult to recruit a large number of respondents. The 2016 UK CF Registry Survey had 224 respondents with CF, of whom 68% were female. Also in 2016, the James Lind Alliance Priority Setting Partnership conducted a survey to elicit opinions about research priorities in CF [20]. This survey involved two stages, that is ‘elicitation’ and ‘prioritisation’, which received respectively 95 and 121 responses from people with CF. Both surveys had additional respondents from other members of the CF community, including parents/guardians and medical professionals.

With patient registry data it is possible to develop personally tailored information on life expectancy and milestones [13]. Receiving personalised information was seen as desirable by many respondents, although not everybody will want it. Therefore, it is important that careful consideration be given as to how this information is delivered. Respondents expressed interest in receiving information via care teams and online. It is important that patients and caregivers be supported to interpret information on estimated survival estimates, together with the caveats and uncertainties involved. An area for further study would be to investigate how receiving information of life expectancy could impact on quality of life and clinical outcomes. In addition to providing information to people with CF and their families and care team, personalised prognostic information could inform decisions surrounding treatment, including listing for transplantation. There is much scope for qualitative work involving patients and clinicians to better understand how they make use of survival data in CF, drawing on insights from the risk communication literature [21,22]. Follow-up work motivated by the outcomes from this study is now underway to assess communication of survival information to adults with CF. It is hoped our results will encourage further qualitative work in this area and help initiate conversations about life expectancy between CF professionals and their patients.

## Supporting information

S1 File The questionnaire (designed using Bristol Online Surveys https://www.onlinesurveys.ac.uk/) as it appeared online, in pdf form. (PDF)

S2 File Tables A-F and Figure A. (PDF)

S3 File The information sheet summarising the results, as sent out to respondents who supplied their email address and as publicised via various routes. (PDF)

## References

[1] Elborn JS, Shale DJ, Britton JR. Cystic fibrosis: current survival and population estimates to the year 2000. *Thorax* 1991; 46:881–885. 1792634 10.1136/thx.46.12.881 PMC463492

[2] Dodge J, Lewis P, Stanton M, Wilsher J. Cystic fibrosis mortality and survival in the UK: 1947–2003. *Eur Respir J*. 2007; 29: 522–526. 10.1183/09031936.00099506 17182652

[3] Jackson AD, Daly L, Jackson AL, Kelleher C, Marshall BC, Quinton HB, et al Validation and use of a parametric model for projecting cystic fibrosis survivorship beyond observed data: a birth cohort analysis. *Thorax* 2011; 66: 674–679. 10.1136/thoraxjnl-2011-200038 21653925 PMC3142345

[4] Cystic Fibrosis Trust. Cystic Fibrosis Registry Report 2016. https://www.cysticfibrosis.org.uk/the-work-we-do/uk-cf-registry/reporting-and-resources.

[5] Buzzetti R, Salvatore D, Baldo E, Forneris MP, Lucidi V, Manunza D, et al An overview of international literature from cystic fibrosis registries: 1. Mortality and survival studies in cystic fibrosis. *J Cyst Fibros* 2009; 8: 229–237. 10.1016/j.jcf.2009.04.001 19419909

[6] McCarthy C, O’Carroll O, Franciosi A, McElvaney N. *Factors Affecting Prognosis and Prediction of Outcome in Cystic Fibrosis Lung Disease*. In: Cystic Fibrosis in the Light of New Research (Editor: Wat D), ISBN: 978-953-51-2152-7, InTech, 10.5772/60899 2015 Available from: http://www.intechopen.com/books/cystic-fibrosis-in-the-light-of-new-research/factors-affecting-prognosis-and-prediction-of-outcome-in-cystic-fibrosis-lung-disease.

[7] MacNeill S. *Epidemiology of Cystic Fibrosis* In: Hodson and Geddes’ Cystic Fibrosis, 4^th^ Edition Editors: Bush A, Bilton D, Hodson M. CRC Press 2015.

[8] Liou TG, Adler FR, Fitzsimmons SC, Cahill BC, Hibbs JR, Marshall BC. Predictive 5-year survivorship model of cystic fibrosis. Am J Epidemiol 201; 153: 345–352. 11207152 10.1093/aje/153.4.345 PMC2198936

[9] Aaron SD, Stephenson AL, Cameron DW, Whitmore GA. A statistical model to predict one-year risk of death in patients with cystic fibrosis. Journal of Clinical Epidemiology 2015; 68: 1336–1345. 10.1016/j.jclinepi.2014.12.010 25655532

[10] Nkam L, Lambert J, Latouche A, Bellis G, Burgel PR, Hocine MN, et al A 3-year prognostic score for adults with cystic fibrosis. JCF 2017; 119: 682–689. 10.1016/j.jcf.2017.03.004 28330773

[11] Keogh RH, Stanojevic S. A guide to interpreting estimated median age of survival in cystic fibrosis patient registry reports. J Cystic Fibros 2017; 17: 213–217. 10.1016/j.jcf.2017.11.014 PMC5885986 29398488

[12] Keogh RH, Szczesniak R, Taylor-Robinson D, Bilton D. Up-to-date and projected estimates of survival for people with cystic fibrosis using baseline characteristics: A longitudinal study using UK patient registry data. J Cystic Fibros 2017; 17: 218–227. 10.1016/j.jcf.2017.11.019 PMC5885983 29311001

[13] Keogh RH, Seaman SR, Barrett J, Taylor-Robinson D, Szczesniak R. Dynamic prediction of survival in cystic fibrosis: A landmarking analysis using UK patient registry data. Epidemiology 2018; 30: 29–37. 10.1097/EDE.0000000000000920 PMC6276867 30234550

[14] Eysenbach G. Improving the quality of Web surveys: the checklist for Reporting Results of Internet E-Surveys (CHERRIES). J Med Internet Res 2004; 6: e34 10.2196/jmir.6.3.e34 15471760 PMC1550605

[15] Cystic Fibrosis Trust website. “Challenging topics: statistics and life expectancy.” https://www.cysticfibrosis.org.uk/news/life-expectancy#. Accessed 4 December 2017.

[16] Braun V, Clarke V. Using thematic analysis in psychology. Qualitative Research in Psychology 2006; 3: 77–101.

[17] Cystic Fibrosis Trust website. https://www.cysticfibrosis.org.uk/news/candid-views-on-life-expectancy. Accessed 4 December 2017.

[18] Ruth Keogh blog. http://blogs.lshtm.ac.uk/ruthkeogh/. Accessed 4 December 2017.

[19] UK CF Registry Survey Report. https://cms.cysticfibrosis.org.uk//the-work-we-do/uk-cf-registry/reporting-and-resources. Accessed 4 December 2017.

[20] Rowbotham NJ, Smith S, Leighton PA, Rayner OC, Gathercole K, Elliott ZC, et al The top 10 research priorities in cystic fibrosis developed by a partnership between people with CF and healthcare providers. *Thorax* 2018; 73: 388–390. 10.1136/thoraxjnl-2017-210473 28778919 PMC5870449

[21] O’Connor AM, Legare F, Stacey D. Risk communication in practice: the contribution of decision aids. *BMJ* 2003; 327: 736–740. 10.1136/bmj.327.7417.736 14512487 PMC200814

[22] Rakow T, Wright RJ, Bull C, Spiegelhalter DJ. Simple and Multistate Survival Curves: Can People Learn to Use Them? *Med Decis Making* 2012; 32: 792–804. 10.1177/0272989X12451057 22753419

