# Supplementary material for: Results from an online survey of adults with cystic fibrosis: Accessing and using life expectancy information
Source: PLoS One. 2019 Apr 12;14(4):e0213639. doi: 10.1371/journal.pone.0213639 (PMC6461271; doi:10.1371/journal.pone.0213639)
Supplement: S1 File — (PDF) [file pone.0213639.s001.pdf]

Online survey to gain understanding of what people with cystic fibrosis aged 16+ would like to learn about their life expectancy and other outcomes

---

Introduction to the researcher and the aims of this questionnaire

About this questionnaire

Who should complete this questionnaire?

This questionnaire is for people with CF who are aged 16 or older. I kindly request that you do not complete the questionnaire if you are aged under 16 or if you do not have CF.

What does it involve and how long will it take?

The questionnaire is anonymous. It has 15 questions (including multiple choice and text-based answers) and should take around 10 minutes to complete.

When can I find out a summary of the results?

A summary of the responses to the questionnaire will be made available by 1st December 2016 at http://blogs.lshtm.ac.uk/ruthkeogh/cf-online-questionnaire-results/, via a link on the Cystic Fibrosis Trust website and via the Cystic Fibrosis Trust Newsletter.

About the researcher

My name is Dr Ruth Keogh and I am a statistician and Senior Lecturer

in Medical Statistics at the London School of Hygiene and Tropical Medicine. I am undertaking research which focuses on the use of state-of-the-art statistical methods, and development of some new methods, to gain understanding about the life expectancy of people with CF. In particular I am working on developing more personalised information about life expectancy for people with CF, their families and friends, doctors and CF care team. I also wish to contribute to improving how information on life expectancy is presented to people with CF.

As part of my research I would like to understand better the extent to which people with CF would like to know about their life expectancy, how people with CF discover and use information about their life expectancy and what people with CF might like to know about it which is currently not available to them. That is the aim of this questionnaire. You can see the full protocol for this questionnaire and also get more information here: http://blogs.lshtm.ac.uk/ruthkeogh/cf-online-questionnaire/

The information which will ultimately result from my research will be used in three main ways:

1. To provide people with CF an answer to the question "Given my current and historical health status, and given I have reached my current age, how long might I expect to live?".
2. To provide better information for doctors about what a patient's current health status could mean for their future needs, which may help to inform treatment decisions.
3. To gain understanding of which measures of health status are most associated with good prognosis, and whether the importance of different measures changes with age.

I am very grateful for you taking the time to complete this questionnaire. The results will be extremely valuable and helpful for my future research and will contribute to improving how information on life expectancy and other outcomes is presented to people with CF.

Thank you!

Ruth

Funding and support

- Ruth Keogh is funded by a Medical Research Council Fellowship.
- This work is also linked to funding by a Strategic Research Centre grant from the Cystic Fibrosis Trust ("*Cystic Fibrosis Epidemiological Network (CF-EpiNet) – Harnessing Data to Improve Lives*"), on which Ruth Keogh is a co-investigator.
- This questionnaire is also supported by the Cystic Fibrosis Trust, who are helping in the distribution of the questionnaire and the publication of the results.

Elibility criteria

This questionnaire is designed to be completed only by people with CF who are aged 16 or older. I kindly request that you do not complete this questionnaire if you are aged under 16 or do not have CF.

1. Are you a person with CF who is also aged 16 or older?

- Yes
- No

Ethics and consent

By completing this questionnaire you consent to your responses being used to produce a summary of the results, which will be published in a report, a summary information sheet, and articles in academic journals.

The questionnaire is anonymous. Text responses will be summarised so that they do not enable individuals to be identified. No individual text responses will be reproduced directly in the results summary.

This project has been approved by the London School of Hygiene and Tropical Medicine Research Ethics Committee.

Support

If you find yourself upset by any of the issues raised by this questionnaire please be aware that support is available from the following sources:

- From your care team. Please contact your care team at your CF Centre to arrange an appointment with a psychologist or other care team member.
- From the Cystic Fibrosis Trust Helpline. This confidential helpline offers general advice, support and information on any aspect of cystic fibrosis from a friendly and knowledgeable person. The helpline is open 9am to 5pm, Monday to Friday, as well as offering voicemail and email response services. The Cystic Fibrosis Trust Helpline can be contacted by email (helpline@cysticfibrosis.org.uk) or phone (0300 373 1000 or 020 3795 2184).

CF Centres and the Cystic Fibrosis Trust Helpline have been made aware of this questionnaire.

About you

2. What is your sex?

- Male
- Female
- Prefer not to say

3. How old are you?

4. What of the following best describes your current employment status?

- Full-time employment
- Part-time employment
- Self-employed
- Student
- Homemaker
- Disabled
- Unemployed
- Retired
- Other

4.a. If you selected Other, please specify:

5. Do you live in the UK?

- ○ Yes
- ○ No

6. Which of the following best describes your living arrangements?

- ○ Living at home with parents or other close family relatives or guardians
- ○ Living with a spouse or partner
- ○ Living with friends or siblings
- ○ Living alone
- ○ Other

6.a. If you selected Other, please specify:

7. Do you have, or have you ever had, any siblings?

- ○ Yes
- ○ No

7.a. Which of the following describes your siblings? *You can select more than one answer here.*

- [ ] I have one or more siblings who are living and who have CF
- [ ] I have one or more siblings who are living and who do NOT have CF
- [ ] I have one or more siblings who have died and who had CF
- [ ] I have one or more siblings who have died and who did NOT have CF
- [ ] Other

7.a.i. If you selected Other, please specify:

Whether and how you currently find information about life expectancy

8. Has your doctor/CF team ever provided you with information about your life expectancy as part of your routine care?

- Yes
- No
- Not sure

8.a. Regarding your answer "Yes", how beneficial did you find this information, in terms of whether you found the information interesting or useful to know?

- Not at all beneficial
- Somewhat beneficial
- Very beneficial

9. Have you ever actively sought information about your life expectancy from your doctor/CF team?

- Yes, and I received some information from them
- Yes, but I did not receive any information from them
- No
- Not sure

9.a. Regarding your answer "Yes, and I received some information from them", how beneficial did you find this information, in terms of whether you found the information interesting or useful to know?

- Not at all beneficial
- Somewhat beneficial
- Very Beneficial

9.b. Regarding your answer "No", do you think there will be a time when you will want more information about your life expectancy and, if so, for what

purposes? *You can select more than one answer here.*

- ☐ No
- ☐ Perhaps: just for general information
- ☐ Perhaps: in planning your education
- ☐ Perhaps: in planning your career path
- ☐ Perhaps: in planning meeting a partner
- ☐ Perhaps: in planning your family
- ☐ Perhaps: in choosing how you spend your leisure time
- ☐ Perhaps: to help make decisions or have discussions jointly with your CF specialist team on future treatments
- ☐ Perhaps: to help plan strategies for maintaining as best health as possible (e.g. your exercise programme, physical activity schedules)
- ☐ Perhaps: to help manage mentally/psychologically your current health status
- ☐ Perhaps: in making other life plans

9.c. Regarding your answer "Not sure", do you think there will be a time when you will want more information about your life expectancy and, if so, for what purposes? *You can select more than one answer here.*

- ☐ No
- ☐ Perhaps: just for general information
- ☐ Perhaps: in planning your education
- ☐ Perhaps: in planning your career path
- ☐ Perhaps: in planning meeting a partner
- ☐ Perhaps: in planning your family
- ☐ Perhaps: in choosing how you spend your leisure time
- ☐ Perhaps: to help make decisions or have discussions jointly with your CF specialist team on future treatments
- ☐ Perhaps: to help plan strategies for maintaining as best health as possible (e.g. your exercise programme, physical activity schedules)
- ☐ Perhaps: to help manage mentally/psychologically your current health

status

- [ ] Perhaps: in making other life plans

10. Have you ever actively sought information about your life expectancy from any of the following other sources? *You can select more than one answer here.*

- [ ] Reports from the Cystic Fibrosis Trust/the Cystic Fibrosis Trust website
- [ ] Research literature
- [ ] Patient websites/forums
- [ ] Other internet sites
- [ ] Other people
- [ ] Other sources
- [ ] None of these

10.a. Regarding your answer **"Reports from the Cystic Fibrosis Trust/the Cystic Fibrosis Trust website"**, how beneficial did you find this information, in terms of whether you found the information interesting or useful to know?

○ Not at all beneficial ○ Somewhat beneficial ○ Very beneficial

10.b. Regarding your answer **"Research literature"**, how beneficial did you find this information, in terms of whether you found the information interesting or useful to know?

○ Not at all beneficial ○ Somewhat beneficial ○ Very beneficial

10.c. Regarding your answer **"Patient websites/forums"**, how beneficial did you find this information, in terms of whether you found the information interesting or useful to know?

○ Not at all beneficial ○ Somewhat beneficial ○ Very beneficial

10.d. Regarding your answer **"Other internet sites"**, how beneficial did you find this information, in terms of whether you found the information interesting or useful to know?

- ○ Not at all beneficial
- ○ Somewhat beneficial
- ○ Very beneficial

10.e. Regarding your answer **"Other people"**, how beneficial did you find this information, in terms of whether you found the information interesting or useful to know?

- ○ Not at all beneficial
- ○ Somewhat beneficial
- ○ Very beneficial

10.f. Regarding your answer **"Other sources"**, how beneficial did you find this information, in terms of whether you found the information interesting or useful to know?

- ○ Not at all beneficial
- ○ Somewhat beneficial
- ○ Very beneficial

10.g. If you wish, please provide any information here about other sources you have used. Please also provide any information here about what you have found particularly beneficial or not about the different sources you have used. *Optional*

10.h. Regarding your answer **"None of these"**, why have you not sought information about your life expectancy? *You can select more than one answer here.*

Please select at least 1 answer(s).

- ☐ Because you feel you have received most or all of the information you

would like from your doctor/CF team

- ☐ Because you don’t want to know
- ☐ Because you feel the information available will not be relevant and/or useful to you
- ☐ Other

10.h.i. If you selected Other, please specify:

11. How do you use, or how have you used in the past, any information which you have learned about your life expectancy, either from your doctor/CF care team or from other sources? *You can select more than one answer here.*

- ☐ Not much
- ☐ Just for general information
- ☐ In planning your education
- ☐ In planning your career path
- ☐ In planning meeting a partner
- ☐ In planning your family
- ☐ In choosing how you spend your leisure time
- ☐ To help make decisions or have discussions jointly with your CF specialist team on future treatments
- ☐ To help plan strategies for maintaining as best health as possible (eg. your exercise programme, physical activity schedules)
- ☐ To help manage mentally/psychologically your current health status
- ☐ In making other life plans
- ☐ I have never received any information about my life expectancy

The potential for more personalised information on life expectancy

12. Would you like to be able to access more personalised information about your life expectancy? The personalised information on which this is based could include, for example, your FEV1% predicted and how this is changing as you get older, your weight, the treatments you are using, whether you have received an organ transplant, as well as more intrinsic features such as your gender and your genetics.

- ○ Yes
- ○ No
- ○ Not sure

12.a. Regarding your answer "Yes", how do you think you would prefer to receive this information? *You can select more than one answer here.*

- ☐ At the clinic from my doctor/CF care team
- ☐ By myself, for example via an online tool
- ☐ Other

12.a.i. If you selected Other, please specify:

13. One of the aims of my research is to provide more personalised information on your life expectancy which can be *updated* as you get older to take into account up-to-date information about your health status. Would you find such information useful as an indicator of how you are doing, including how you are doing relative to other people the same age as you (even if you are not

specifically interested in your life expectancy)?

- Yes
- No
- Not sure

13.a. Regarding you answer "Yes", how do you think you would prefer to receive this information? *You can select more than one answer here.*

- [ ] At the clinic from my doctor/CF care team
- [ ] By myself, for example via an online tool
- [ ] Other

13.a.i. If you selected Other, please specify:

14. Would you be interested in how long it might be until you reach other milestones, in addition to or instead of your overall life expectancy? For example reaching a level of FEV1% predicted, having a transplant, or acquiring chronic pseudomonas.

- Yes
- No
- Not sure

14.a. Regarding your answer "Yes", what milestones would you be interested in? *You could mention those listed above and/or any other milestones.*

15. Is there any information you would like to access about your life expectancy or about reaching other milestones which has not been covered here, and if so what?

Questionnaire results

A summary of the responses to the questionnaire will be made available by 1st December 2016 at http://blogs.lshtm.ac.uk/ruthkeogh/cf-online-questionnaire-results/, via a link on the Cystic Fibrosis Trust website and via the Cystic Fibrosis Trust Newsletter.

16. If you would like to receive an information sheet summarising the results by email when they become available, please provide your email address below. Please be assured that your email address will be used only for the purpose of sending this information, will be stored securely by the investigator and will not be passed on to anyone else.

Please enter a valid email address.

Reminder of support

If you find yourself upset by any of the issues raised by this questionnaire please be aware that support is available from the following sources:

- From your care team. Please contact your care team at your CF Centre to arrange an appointment with a psychologist or other care team member.
- From the Cystic Fibrosis Trust Helpline. This confidential helpline offers general advice, support and information on any aspect of cystic fibrosis from a friendly and knowledgeable person. The helpline is open 9am to 5pm, Monday to Friday, as well as offering voicemail and email response services. The Cystic Fibrosis Trust Helpline can be contacted by email (helpline@cysticfibrosis.org.uk) or phone (0300 373 1000 or 020 3795 2184).

CF Centres and the Cystic Fibrosis Trust Helpline have been made aware of this questionnaire.

Thank you

Many thanks for completing this questionnaire.

Key for selection options

3 - How old are you?

16
17
18
19
20
21
22
23
24
25
26
27
28
29
30
31
32
33
34
35
36
37
38
39
40
41
42
43
44
45
