# Supplementary material for: Results from an online survey of adults with cystic fibrosis: Accessing and using life expectancy information
Source: PLoS One. 2019 Apr 12;14(4):e0213639. doi: 10.1371/journal.pone.0213639 (PMC6461271; doi:10.1371/journal.pone.0213639)
Supplement: S2 File — (PDF) [file pone.0213639.s002.pdf]

Results from an online survey of adults with cystic fibrosis: accessing and using life expectancy information

Supplementary Materials

Ruth H. Keogh[1*], Diana Bilton[2], Rebecca Cosgriff[3], Dominic Kavanagh[3], Oliver Rayner[3], Philip M. Sedgwick[4,5].

[1] Department of Medical Statistics, London School of Hygiene and Tropical Medicine, Keppel Street, London, UK, WC1E 7HT.

[2] Royal Brompton Hospital, Adult Cystic Fibrosis Centre, London, UK, SW3 6NP.

[3] Cystic Fibrosis Trust, One Aldgate, Second floor, London, UK, EC3N 1RE.

[4] Institute for Medical and Biomedical Education, St George's, University of London, Cranmer Terrace, London, UK, SW17 0RE.

[5] South West London Elective Orthopaedic Centre, Epsom and St. Helier University NHS Hospitals, Epsom, KT18 7EG

* Corresponding author

Email: ruth.keogh@lshtm.ac.uk.

Table A: Summary of questions and sub-questions included in the questionnaire, including the type of response and the possible responses for multiple choice questions.

Question number	Question	Possible responses (where applicable)	Type of response
	**Filter question**		
1	Are you a person with CF who is also aged 16 or older?	Yes; No	Single response
	**"About you"**		
2	What is your sex?	Male; Female; Prefer not to say	Single response
3	How old are you?	Drop down menu given with ages 16-99	Single response
4	What of the following best describes your current employment status?	Full-time employment; Part-time employment; Self-employed; Student; Homemaker; Disabled; Unemployed; Retired; Other	Single response
4a	If you selected Other, please specify		Free text
5	Do you live in the UK?	Yes; No	Single response
6	Which of the following best describes your living arrangements?	Living at home with parents or other close family relatives or guardians; Living with a spouse or partner; Living with friends or siblings; Living alone; Other	Single response
6a	If you selected Other, please specify		Free text
7	Do you have, or have you ever had, any siblings?	Yes; No	Single response
7a	Which of the following describes your siblings?	I have one or more siblings who are living and who have CF; I have one or more siblings who are living and who do NOT have CF; I have one or more siblings who have died and who had CF; I have one or more siblings who have died and who did NOT have CF; Other	Multiple response
7a(i)	If you selected Other, please specify		Free text
	**"Whether and how you currently find information about life expectancy"**		
8	Has your doctor/CF team ever provided you with information about your life expectancy as part of your routine care?	Yes; No; Not sure	Single response
8a	Regarding your answer "Yes", how beneficial did you find this information, in terms of whether you found the information interesting or useful to know?	Not at all beneficial; Somewhat beneficial; Very beneficial	Single response
9	Have you ever actively sought information about your life expectancy from your doctor/CF team?	Yes, and I received some information from them; Yes, but I did not receive any information from them; No; Not sure	Single response
9a	Regarding your answer "Yes, and I received some information from them", how beneficial did you find this information, in terms of whether you found the information interesting or useful to know?	Not at all beneficial; Somewhat beneficial; Very beneficial	Single response
9b	Regarding your answer "No", do you think there will be a time when you will want more information about your life expectancy and, if so, for what purposes?	No; Perhaps: just for general information; Perhaps: in planning your education; Perhaps: in planning your career path; Perhaps: in planning meeting a partner; Perhaps: in planning your family; Perhaps: in choosing how you spend your leisure time; Perhaps: to help make decisions or have discussions jointly with your CF specialist team on future treatments; Perhaps: to help plan strategies for maintaining as best health as possible (e.g. your exercise programme, physical activity schedules); Perhaps: to help manage mentally/psychologically your current health status; Perhaps: in making other life plans	Multiple response

9c	Regarding your answer "Not sure", do you think there will be a time when you will want more information about your life expectancy and, if so, for what purposes?	As in 9b	Multiple response
10	Have you ever actively sought information about your life expectancy from any of the following other sources?	Reports from the Cystic Fibrosis; Trust/the Cystic Fibrosis Trust website; Research literature; Patient websites/forums; Other internet sites; Other people; Other sources; None of these;	Multiple response
10a-10f	Regarding your answer **"Reports from the Cystic Fibrosis Trust/the Cystic Fibrosis Trust website" [for each source listed in Question 10]**, how beneficial did you find this information, in terms of whether you found the information interesting or useful to know?	Not at all beneficial; Somewhat beneficial; Very beneficial	Single response
10g	If you wish, please provide any information here about other sources you have used. Please also provide any information here about what you have found particularly beneficial or not about the different sources you have used.		Free text.
10h	Regarding your answer **"None of these"**, why have you not sought information about your life expectancy?	Because you feel you have received most or all of the information you would like from your doctor/CF team; Because you don't want to know; Because you feel the information available will not be relevant and/or useful to you; Other	Multiple response
10h(i)	If you selected Other, please specify:		Free text.
11	How do you use, or how have you used in the past, any information which you have learned about your life expectancy, either from your doctor/CF care team or from other sources?	Not much; Just for general information; In planning your education; In planning your career path; In planning meeting a partner; In planning your family; In choosing how you spend your leisure time; To help make decisions or have discussions jointly with your CF specialist team on future treatments; To help plan strategies for maintaining as best health as possible (eg. Your exercise programme, physical activity schedules); To help manage mentally/psychologically your current health status; In making other life plans; I have never received any information about my life expectancy	Multiple response
	**"The potential for more personalised information on life expectancy"**		
12	Would you like to be able to access more personalised information about your life expectancy? The personalised information on which this is based could include, for example, your FEV1% predicted and how this is changing as you get older, your weight, the treatments you are using, whether you have received an organ transplant, as well as more intrinsic features such as your gender and your genetics.	Yes; No; Not sure	Single response.
12a	Regarding your answer "Yes", how do you think you would prefer to receive this information?	At the clinic from my doctor/CF care team; By myself, for example via an online tool; Other	Multiple response.
12a(i)	If you selected Other, please specify:		Free text.
13	One of the aims of my research is to provide more personalised information on your life expectancy which can be *updated* as you get older to take into account up-to-date information about your health status. Would you	Yes; No; Not sure	Single response.

	find such information useful as an indicator of how you are doing, including how you are doing relative to other people the same age as you (even if you are not specifically interested in your life expectancy)?		
13a	Regarding you answer "Yes", how do you think you would prefer to receive this information?	At the clinic from my doctor/CF care team; By myself, for example via an online tool; Other	Multiple response.
13a(i)	If you selected Other, please specify:		Free text.
14	Would you be interested in how long it might be until you reach other milestones, in addition to or instead of your overall life expectancy? For example reaching a level of FEV1% predicted, having a transplant, or acquiring chronic pseudomonas.	Yes; No; Not sure	Single response.
14a	Regarding your answer "Yes", what milestones would you be interested in? *You could mention those listed above and/or any other milestones*.		Free text.
15	Is there any information you would like to access about your life expectancy or about reaching other milestones which has not been covered here, and if so what?		Free text.

Figure A: Number of questionnaire responses by date.

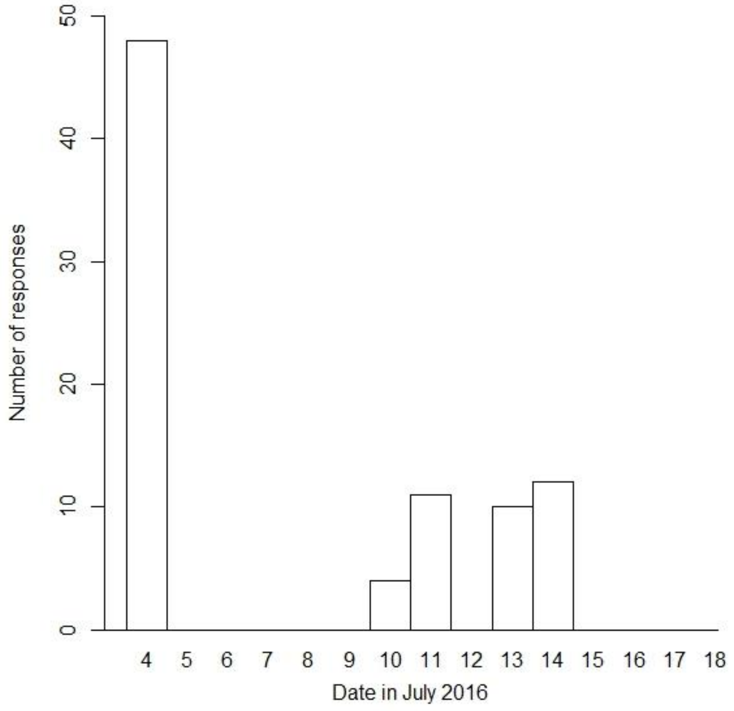

Table B: Information from the Cystic Fibrosis Trust on how many people were exposed to information about the questionnaire via their social media.

**Facebook**	Reach	37,705
	Number of likes	292
	Number of comments	9
	Number of shares	46
	Number of link clicks	832
**Twitter**	Number of impressions	2671
	Number of retweets	4
	Number of likes	5
	Number of clickthroughs	10

Table C: Summary of 7 text responses given to question 10h(i): "Have you ever actively sought information about your life expectancy from any of the following other sources*? [None of these]* Regarding your answer "None of these", why have you not sought information about your life expectancy? *[Other]* If you selected Other, please specify." The responses have been paraphrased.

**1**	Reduced life expectancy is just a part of life with CF and they didn't think about it or preferred not to dwell on death, and that doing so could impact poorly on health if it results in a negative attitude.
**2**	They preferred to focus positively on their care and on living life and doing things they enjoyed.
**3**	Life expectancy is difficult to talk about
**4**	They assumed no one would be able to tell them about it
**5**	They assumed life expectancy depends on unknown future developments in treatment.

Table D: Summary of 18 text responses given to the question (10g): "If you wish, please provide any information here about other sources you have used. Please also provide any information here about what you have found particularly beneficial or not about the different sources you have used.". The responses have been paraphrased.

**1**	Discussions about life expectancy with the CF care team are more emotional than investigating more generic information online. Information obtained from the CF care team can be non-concrete, while information available online can feel more concrete but is not individualised.
**2**	CF is a condition affecting many different aspects of life and not all aspects are perhaps always appreciated by CF care teams. CF is also a varied condition which affects people differently.
**3**	CF affects people differently and it can be difficult to relate the information which is available to your own condition. There is a desire for information on how specific aspects of CF in a specific individual affect life expectancy and a lack of this kind of information currently.
**4**	There is a desire for balanced and honest information on CF for what it is – not making it sound better or worse than it really is.
**5**	Generic information on life expectancy does not apply easily to individuals, especially once a person has reached the 'median survival age'. Information on 'median life expectancy', 'median age at death' and other measures can appear inconsistent and can be confusing. One person specifically remarked that they would like more personalised information.
**6**	Some people with CF accept that their life expectancy is lowered but do not dwell on it and get on with life. Some people have looked at information on life expectancy out of curiosity, but did not find themselves affected by it.
**7**	One respondent recalled having only discovered by chance at a young age that life expectancy was lowered for people with CF.
**8**	Other sources from which people had sought information on life expectancy were: Registry Reports both from the UK and other countries; the latest estimates of life expectancy on the Cystic Fibrosis Trust web site; presentations; Wikipedia; Google; the general media. Some of the information available online is not up to date and in some of the research literature the information on life expectancy is not detailed enough. The Wikipedia article was noted to be good.
**9**	Some have found research literature useful for making a decision about joining the list for lung transplantation.
**10**	There is a recognition that information will always be in some sense 'average' and it is impossible to say for certain what will happen to a person in the future.
**11**	It is important to get a balance of scientific information relating to life expectancy together with information on real life experiences.

Table E: Summary of 17 responses to question 15: "Is there any information you would like to access about your life expectancy or about reaching other milestones which has not been covered here, and if so what?". Four of the 17 responses were "No". General information provided in earlier text responses have also been incorporated into this summary. The responses have been paraphrased.

**1**	How do a range of factors affect life expectancy: diabetes, pancreatic insufficiency, depression, pseudomonas, mycobacterium abscessus, chronic infection, whether you have had a transplant, the age at which chronic infections were acquired, weight loss. Also an interest in how certain factors relate to other measures such as median age at death.
**2**	What are the complications associated with CF-related diabetes and other CF-related diseases?
**3**	How do I compare with other people with CF?
**4**	How are other people in a similar situation to me going about their care and treatments? How long to people spend on their treatments and how does it affect their life expectancy?
**5**	Information on post-transplant survival.
**6**	How does CF progress in terms of functional milestones? Information on whether people tend to experience a gradual decline or a more sudden decline could help people plan better for their future and make more informed decisions about how they want to live their lives.
**7**	How the number of exacerbations people have changed over time.
**8**	To what extent, if at all, can lung function be recovered via treatments? And if a lung function is improved by treatment, is this maintained or does it then return to a lower level?
**9**	What are the effects of current medications? What might the effects of future medications be and how might they improve things?
**10**	The difficulty of making certain decisions and desire for more information on topics in many areas of life: about starting a family and how this could impact on your health; about slowing down in terms of work and whether this could preserve your health for longer; about financial planning and saving for a pension.
**11**	The difficulties and pressures of comparing yourself with people without CF in terms of what you can do, for example by having a job. Also, a desire for information on other issues relating to quality of life, such as being able to do exercise and the amount of time people with CF sleep.
**12**	Period of poor health and the corresponding intensive treatments can cause a great deal of anxiety. But you don't know what will happen in the future. There can be periods of recovery even from a very bad state and doctors can't always predict what will happen.
**13**	The importance of placing information on life expectancy in the correct context and also providing advice alongside this information on what could be done to improve life expectancy.
**14**	Recognition that a dip in lung function, say, may be a one-off and needs to be interpreted in the context of other information. This is something that can be done by the CF care team via their experience and knowledge of the patient, but it could be more difficult to account for in statistical results.
**15**	Some people might find information on life expectancy to be a motivator to try to keep ahead of what is predicted.

Table F: Summary of main questions by sex, age and siblings status. Results shown are "Number (%)". The p-values are from a test (using Fisher's exact test) of whether the responses differed by sex/age/siblings status.

Question	***Sex***		**Age**		**Siblings status**	
	**Men**	**Women**	**Under 30**	**30+**	**has siblings without CF only, or no siblings**	**has siblings with CF**
**8. Has your doctor/CF team ever provided you with information about your life expectancy as part of your routine care?**						
Yes	11 (33.3)	10 (19.2)	12 (30.0)	9 (20.0)	17 (24.6)	4 (25.0)
No	16 (48.5)	40 (76.9)	24 (60.0)	32 (71.1)	45 (65.2)	11 (68.8)
Not sure	6 (18.2)	2 (3.8)	4 (10.0)	4 (8.9)	7 (10.1)	1 (6.3)
p-value	0.016		0.558		1	
**9. Have you ever actively sought information about your life expectancy from your doctor/CF team?**						
Yes	8 (24.2)	15 (28.8)	9 (22.5)	14 (31.1)	21 (30.4)	2 (12.5)
No	25 (75.8)	35 (67.3)	29 (72.5)	31 (68.9)	47 (68.1)	13 (81.3)
Not sure	0 (0)	2 (3.8)	2 (5.0)	0 (0)	1 (1.4)	1 (6.3)
p-value	0.597		0.269		0.139	
**10. Have you ever actively sought information about your life expectancy from any other sources?**						
Yes	9 (27.3)	16 (30.8)	9 (22.5)	16 (35.6)	24 (34.8)	1 (6.3)
No	24 (72.7)	36 (69.2)	31 (77.5)	29 (64.4)	45 (65.2)	15 (93.8)
p-value	0.810		0.236		0.031	
**12. Would you like to be able to access more personalised information about your life expectancy?**						
Yes	23 (69.7)	39 (75.0)	31 (77.5)	31 (68.9)	52 (75.4)	10 (62.5)
No	8 (24.2)	3 (5.8)	4 (10.0)	7 (15.6)	8 (11.6)	3 (18.8)
Not sure	2 (6.1)	10 (19.2)	5 (12.5)	7 (15.6)	9 (13.0)	3 (18.8)
p-value	0.019		0.687		0.485	
**13. Would you find personalised information on life expectancy useful as an indicator of how you are doing, including how you are doing relative to other people the same age as you (even if you are not specifically interested in your life expectancy)?**						
Yes	23 (69.7)	47 (90.4)	37 (92.5)	33 (73.3)	57 (82.6)	13 (81.3)
No	8 (24.2)	0 (0)	2 (5.0)	6 (13.3)	6 (8.7)	2 (12.5)
Not sure	2 (6.)	5 (9.6)	1 (2.5)	6 (13.3)	6 (8.7)	1 (6.3)
p-value	<0.001		0.079		0.863	
**14. Would you be interested in how long it might be until you reach other milestones, in addition to or instead of your overall life expectancy?**						
Yes	15 (45.5)	31 (59.6)	21 (52.5)	25 (55.6)	38 (55.1)	8 (50.0)
No	11 (33.3)	9 (17.3)	12 (30.0)	8 (17.8)	16 (23.2)	4 (25.0)
Not sure	7 (21.2)	12 (23.1)	7 (17.5)	12 (26.7)	15 (21.7)	4 (25.0)
p-value	0.250		0.366		0.934	
