# Supplementary material for: Results from an online survey of adults with cystic fibrosis: Accessing and using life expectancy information
Source: PLoS One. 2019 Apr 12;14(4):e0213639. doi: 10.1371/journal.pone.0213639 (PMC6461271; doi:10.1371/journal.pone.0213639)
Supplement: S3 File — (PDF) [file pone.0213639.s003.pdf]

What would

PEOPLE WITH CYSTIC FIBROSIS

aged 16+ like to learn about

THEIR LIFE EXPECTANCY

and other outcomes?

results from an online survey

THANK YOU
to everyone who took the time to complete this questionnaire. This sheet summarises some of the main findings - you can find more detailed results at **HTTP://TINYURL.COM/CFQ16-RES**

We are grateful for the many detailed text responses that were given; these are extremely useful and enlightening and are summarised in the detailed results. At the end of this information sheet you can find out about how the results will be used.

PLEASE CONTACT RUTH KEOGH IF YOU HAVE ANY COMMENTS OR QUESTIONS:
ruth.keogh@lshtm.ac.uk

ABOUT THE RESPONDENTS

85 people completed the survey, which was open 4th - 18th July 2016

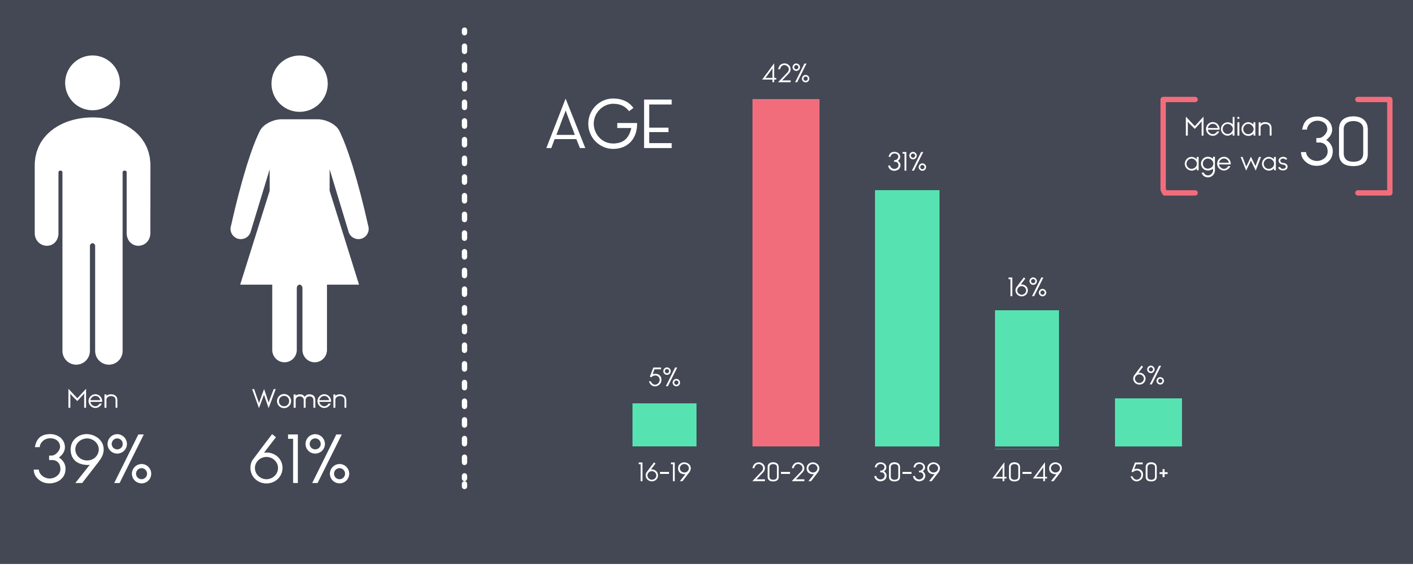

What people said about getting

INFORMATION ON LIFE EXPECTANCY

from doctors or care teams

HAVE YOU ACTIVELY SOUGHT INFORMATION ON LIFE EXPECTANCY FROM YOUR DOCTOR / CF CARE TEAM?

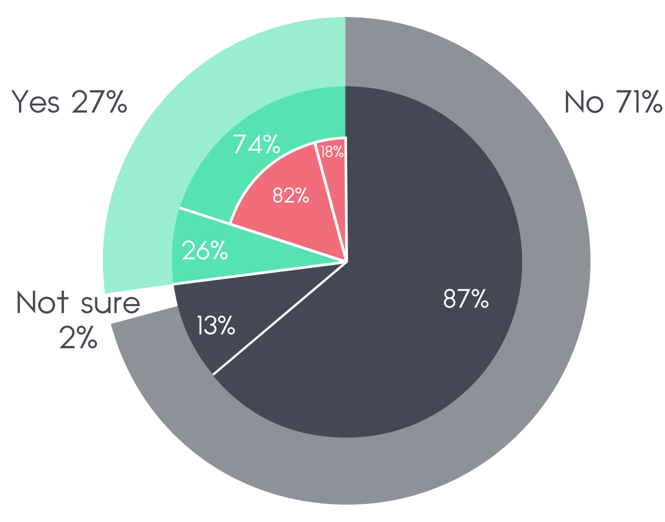

74% of those who sought information actually received some information

82% found that information beneficial

87% of those who said "No" or "Not sure" thought there may be a time in the future when they would want such information

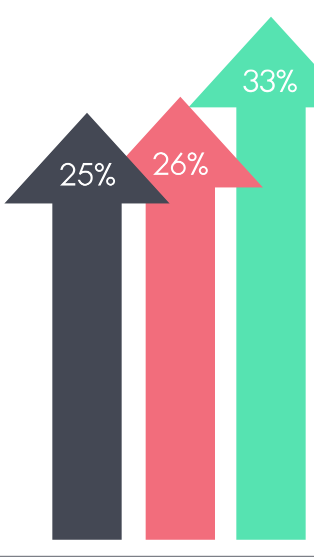

TOP 3 reasons for perhaps wanting this information in the future:

- In making other life plans
- To help plan strategies for maintaining as best health as possible
- To help manage mentally/psychologically your current health status

HAVE YOU EVER BEEN PROVIDED WITH INFORMATION ON LIFE EXPECTANCY FROM YOUR DOCTOR / CF CARE TEAM AS PART OF YOUR ROUTINE CARE?

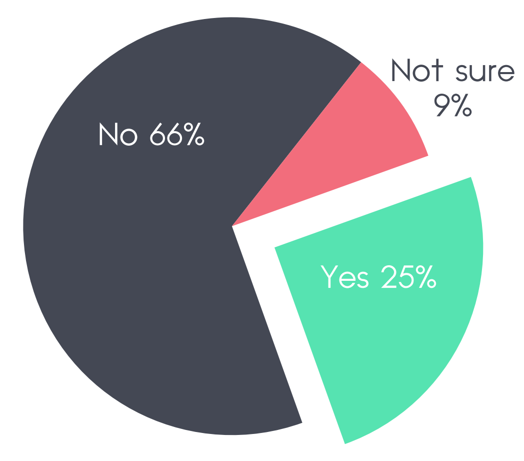

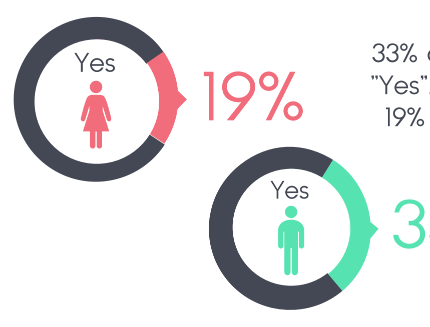

33% of men said "Yes", versus only 19% of women

Of those who received information, 29% found it very beneficial, 52% somewhat beneficial and 19% not at all beneficial

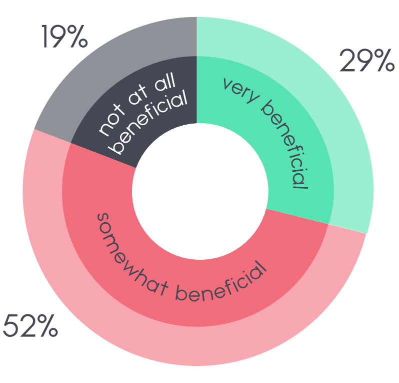

What people said about getting

INFORMATION ON LIFE EXPECTANCY

from other sources

HAVE YOU SOUGHT INFORMATION FROM ANY OF THESE SOURCES?

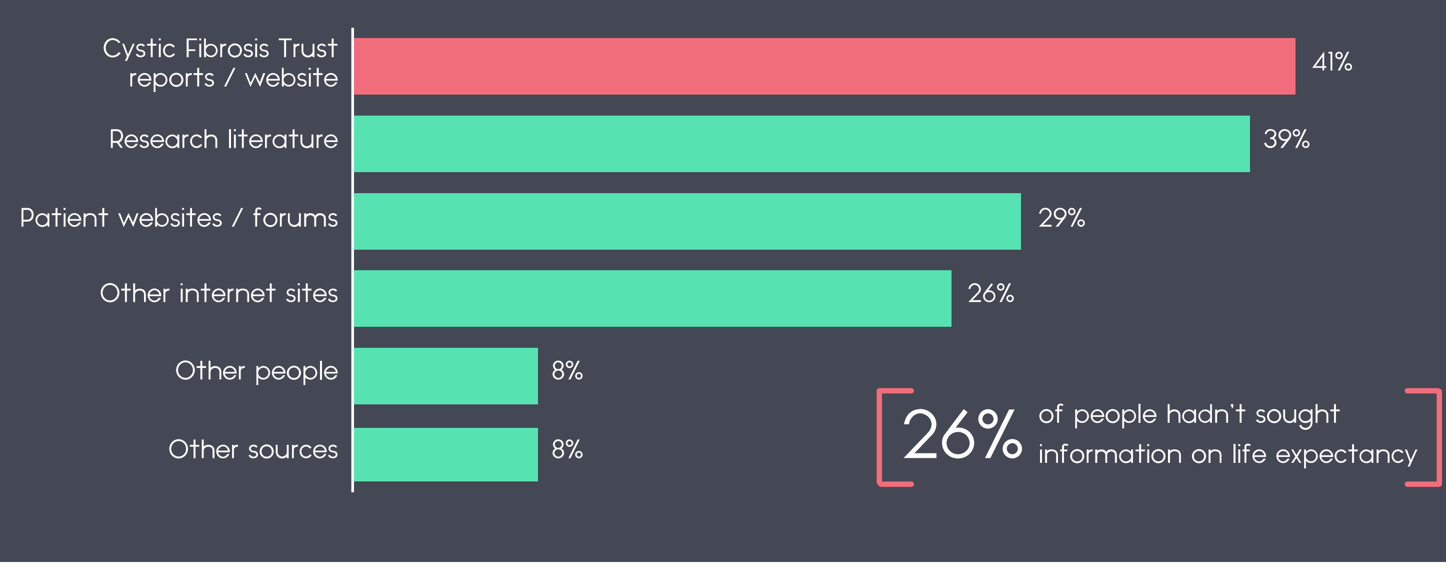

26% of people hadn't sought information on life expectancy

WHAT DO PEOPLE USE INFORMATION ON LIFE EXPECTANCY FOR?

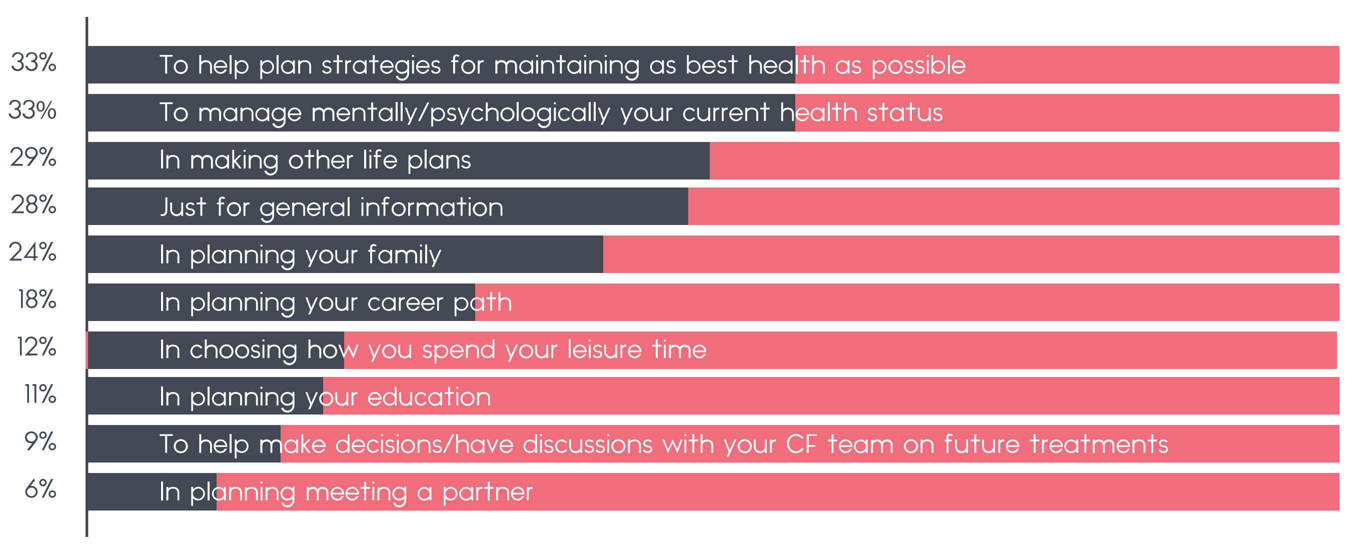

What people said about getting

MORE PERSONALISED INFORMATION

on life expectancy and other outcomes

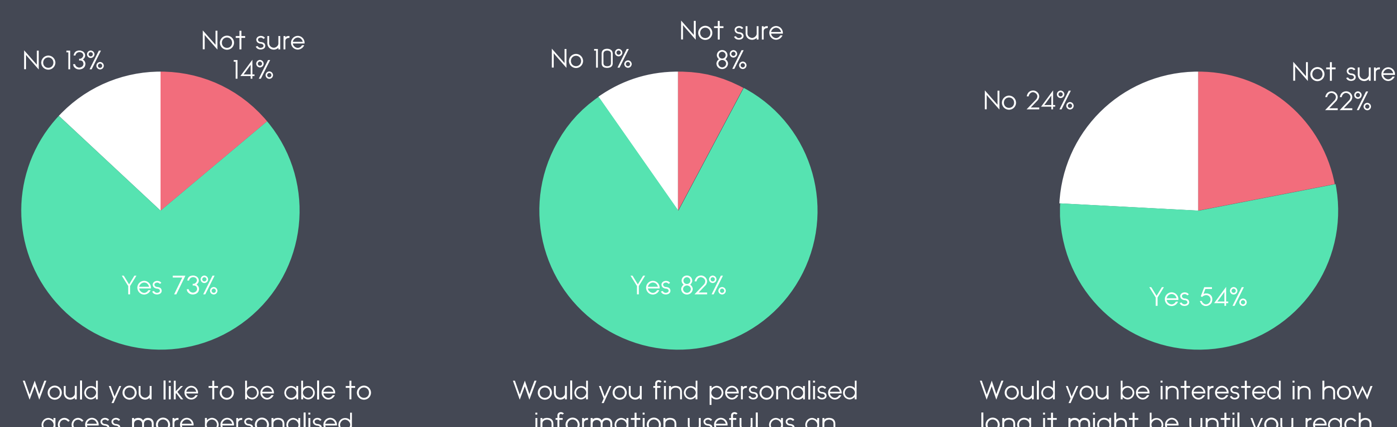

Would you like to be able to access more personalised information about your life expectancy?

Would you find personalised information useful as an indicator of how you are doing, including relative to other people the same age as you?

Would you be interested in how long it might be until you reach other milestones?

The three most commonly mentioned milestones were:

- **63%** Transplant
- **52%** Reaching a certain level of FEV1%
- **28%** Acquiring infections

NEXT STEPS

The results from this survey will be used in a number of ways, including:

- In further work on how best to present information on life expectancy to people with CF and on how such information can be used in a positive way
- To inform the use of data from the UK Cystic Fibrosis Registry in future research
- To inform CF care teams about how people are thinking about issues relating to their life expectancy

This work was carried out by
DR RUTH KEOGH
(London School of Hygiene & Tropical Medicine)

in collaboration with Professor Diana Bilton, Rebecca Cosgriff, Dominic Kavanagh, and Oli Rayner.
Special thanks to Ute Schauberger who designed this information sheet. With thanks to the Cystic Fibrosis Trust for advertising the questionnaire, and also to individuals and cf/Aware for promoting it on social media.

LONDON SCHOOL of HYGIENE & TROPICAL MEDICINE

Cystic Fibrosis Trust
